# A Systematic Review of Stair Fall Risk Assessment Instruments in Older Adults

**DOI:** 10.1016/j.arrct.2026.100668

**Published:** 2026-06-28

**Authors:** Taychapat Makkong, David Brienza

**Affiliations:** a Department of Rehabilitation Science and Technology, School of Health and Rehabilitation Sciences, University of Pittsburgh, Pittsburgh, PA; b Department of Bioengineering, Swanson School of Engineering, University of Pittsburgh, Pittsburgh, PA

**Keywords:** Accidental falls, Aged, Measurement properties, Older adult, Psychometrics, Rehabilitation, Rehabilitation assessment, Risk assessment, Stair climbing

## Abstract

**Objective:**

To systematically identify and critically appraise stair-related fall-risk assessment instruments for older adults and summarize evidence on their measurement properties and clinical applicability.

**Data Sources:**

PubMed, CINAHL, Web of Science, and Cochrane Library were searched from inception through October 2025. Reference lists of included studies and relevant reviews were also screened.

**Study Selection:**

Studies were eligible if they included adults aged 60 years or older and evaluated stair-related assessment instruments with at least 1 reported psychometric property. Studies of general fall-risk tools without a stair-specific component, biomechanical studies without a structured assessment instrument, and nonoriginal publications were excluded.

**Data Extraction:**

Two reviewers independently screened studies, reviewed full texts, and extracted data using a standardized form. Methodological quality and measurement property findings were evaluated using COnsensus-based Standards for the selection of health Measurement INstruments Risk of Bias standards and criteria for good measurement properties.

**Data Synthesis:**

After deduplication, 855 records were screened, 60 full-text articles were assessed, and 10 studies, including 1778 participants, were included. Participants had mean ages ranging from 61.4 to 82.7 years, with reported ages from 65 to 97 years. Studies were conducted in community, clinical, outpatient rehabilitation, and research settings. Instruments were primarily performance-based stair tests, along with 1 clinician-rated observational tool and self-report measures of stair self-efficacy. Reliability and construct validity were the most frequently examined and most consistently supported measurement properties, particularly among performance-based and selected observational instruments. Evidence for predictive validity and responsiveness was limited, with relatively stronger support for stair-negotiation time–based measures. Measurement error was commonly reported but frequently judged indeterminate. No instrument demonstrated comprehensive evaluation across all major measurement properties.

**Conclusions:**

Stair-related assessment instruments for older adults show uneven psychometric support. Current evidence most strongly supports selected performance-based and observational measures as complementary components of multifactorial fall-risk assessment rather than stand-alone tools for prognosis or longitudinal monitoring.

Stair-related falls represent a clinically important subset of falls in older adults because they are associated with substantial injury severity, including fractures, traumatic brain injury, and mortality. Stair negotiation is a demanding mobility task that requires coordination of strength, postural control, sensory input, visual guidance, and attention. Age-related changes in these systems may increase instability during stair ascent and descent, underscoring the need for assessment approaches that address stair-specific risk rather than general fall-risk alone.^1^, ^2^, ^3^

Despite the prevalence and severity of stair falls, research and clinical assessment have primarily focused on general fall-risk evaluation rather than task-specific conditions. Commonly used instruments, such as the Berg Balance Scale, Timed Up and Go Test, and Functional Reach Test, assess general balance and mobility but do not directly capture the biomechanical, cognitive, visual, psychological, and environmental demands of stair use. Recent evidence indicates that no single gait, balance, or functional mobility measure can predict fall risk in older adults with high certainty, although gait speed may contribute useful information as part of a comprehensive evaluation.^4^, ^5^, ^6^, ^7^ Systematic reviews of fall-risk tools have provided valuable information on validity and reliability,^8^^,^^9^ yet these reviews indicate that most tools are designed for level-ground assessment and rarely address stair negotiation. As a result, clinicians and researchers lack validated instruments to identify individuals specifically at risk of stair-related falls.

Stair ascent and descent differ substantially from walking on level ground. Negotiating stairs requires greater joint range of motion and higher muscle moments, particularly at the knee and ankle.^10^ Older adults often adopt compensatory movement strategies, such as reduced propulsion forces and slower step clearance, to maintain stability during stair use.^11^ Cognitive and visual control also influence stair performance. Gaze behavior and attention affect foot-placement accuracy, and distractions or dual tasks can impair stair safety.^12^ Psychological factors may also influence stair negotiation. Concerns about falling have been associated with slower stair-negotiation time in community-dwelling older adults, whereas confidence and visual behavior during stair walking may affect foot placement, movement planning, and willingness to use stairs.^1^^,^^12^^,^^13^ These findings highlight that stair negotiation depends on the integration of sensory, motor, and cognitive control systems, distinguishing it from general balance tasks.

Few assessment instruments have been developed to measure stair-related fall risk. Environmental hazards, such as poor lighting, irregular riser heights, and inadequate handrails, interact with age-related physical limitations; however, these factors are rarely incorporated into fall-risk evaluations.^14^ Newer tools, including the Step Test Evaluation of Performance on Stairs (STEPS), have been developed to assess stair function; however, their psychometric evidence remains limited.^15^ Measures of stair-climbing capacity also reflect lower-limb power and functional performance, suggesting that task-specific instruments may be better predictors of fall risk than general tests.^16^

Existing systematic reviews have summarized general fall-risk assessments, but evidence for stair-specific instruments has not been systematically synthesized. This gap is clinically important because stair negotiation reflects the interaction of individual, biological, social, economic, and environmental risk factors emphasized in the World Health Organization’s Step Safely framework. Performance on stairs may be influenced by physical capacity, confidence, attention, visual control, handrail availability, lighting, and stair design. Therefore, a focused synthesis is needed to clarify the reliability, validity, responsiveness, measurement error, and clinical applicability of stair-related assessment tools and to determine which instruments have sufficient psychometric support for clinical or research use.^6^, ^7^, ^8^, ^9^^,^^17^

Given the high incidence and consequences of stair falls, the complex physical, cognitive, psychological, visual, and environmental demands of stair negotiation, and the lack of a comprehensive synthesis of stair-specific assessment instruments, a systematic review is warranted. This systematic review aims to critically appraise available stair-related fall-risk assessment instruments for older adults and synthesize current evidence on their reliability, validity, responsiveness, measurement error, and practical applicability across community, clinical, home, and research settings.

## Methods

### Reporting framework

This systematic review was conducted and reported in accordance with the Preferred Reporting Items for Systematic Reviews and Meta-Analyses (PRISMA) guidelines, including transparent documentation of study selection and narrative synthesis when quantitative pooling was not appropriate.^18^ This systematic review was prospectively registered in the International Prospective Register of Systematic Reviews (PROSPERO); CRD420261295801. The University of Pittsburgh Institutional Review Board determined that the activity does not involve human subjects’ research as defined by DHHS and FDA regulations.

### Information sources and search strategy

A comprehensive literature search was conducted to identify studies evaluating stair-specific fall-risk assessment instruments among older adults. Electronic databases searched included PubMed, CINAHL, Web of Science, and the Cochrane Library. The final search was performed in October 2025. The search strategy combined controlled vocabulary and free-text keywords related to stair use, falls, assessment instruments, and older adults. Core concepts included variations of stair/steps, fall risk, assessment/instrument/test, and older adults. Reference lists of included studies and relevant review articles were manually screened to identify additional eligible studies. The full electronic search strategies for PubMed, CINAHL, Web of Science, and the Cochrane Library, including all search terms, limits, and filters, are provided in supplemental appendix 1.

### Eligibility criteria

#### Inclusion criteria

Studies were included if they met all of the following criteria:

Population: community-dwelling or institutionalized participants aged 60 years or older, regardless of health status (healthy, frail, or with comorbidities). Mixed-age studies were included only if data for participants aged 60 years or older were reported separately or if the sample mean age was ≥60.

Focus of assessment: studies describing, evaluating, or validating instruments designed to assess stair-related fall risk, stair performance, or stair-negotiation ability in any context. Eligible domains included physical, cognitive, psychological, sensory/visual, and environmental factors. Studies on multidimensional instruments were included only when they included a distinct stair-related item or subscale with explicit scoring criteria and reported at least 1 measurement property, such as reliability, validity, responsiveness, measurement error, or predictive ability. Eligible instruments included performance-based tests, clinician-rated observational tools, self-report measures, and environmental assessment tools in which stair negotiation was a primary construct or a clearly defined, explicitly scored component of the assessment.

Measurement properties: studies were eligible if they reported at least 1 psychometric or measurement property, including reliability, validity, measurement error, responsiveness, sensitivity, specificity, or predictive ability.

Study design: original, peer-reviewed research (eg, cross-sectional, longitudinal, experimental, validation).

Language and accessibility: English-language, full-text available.

Setting: any setting (clinical, community, home, or research) in which stair-related performance or fall risk in older adults was assessed.

#### Exclusion criteria

Studies were excluded if they did not meet all the inclusion criteria or met any of the following exclusion criteria:

Instrument: assessed general fall-risk or level-ground mobility instruments without a stair-specific component; or assessed instruments lacking reported psychometric evidence or that were not validated for older adults. Included incidental stair-related content (eg, a single unexamined item within a broader tool) or if the instrument was not intended to assess stair-related function or risk.

Publication type: publications were reviews, editorials, commentaries, protocols, conference abstracts, unpublished theses, or unpublished dissertations.

Study type: was a simulation or biomechanical modeling study without human participant data.

Outcome focus: the study focused solely on biomechanical parameters (eg, kinematics, kinetics, electromyography) and did not evaluate a structured assessment instrument.

### Selection process

All records retrieved from the database searches were imported into reference management software, and duplicates were removed. Title and abstract screening were conducted independently by 2 reviewers using the predefined eligibility criteria. Studies considered potentially eligible by either reviewer were retained for full-text assessment. Full-text articles were then reviewed independently by 2 reviewers against the inclusion and exclusion criteria. Discrepancies were resolved through discussion until consensus was reached.

### Data collection process and data items

Data were extracted by one reviewer using a standardized form and checked by a second reviewer for accuracy and completeness. Discrepancies were resolved through discussion. Extracted items included study characteristics, participant demographics, setting, instrument characteristics, outcome measures, and reported measurement properties. When multiple publications reported data from the same study, information was extracted and synthesized at the study level to avoid duplication.

### COnsensus-based Standards for the selection of health Measurement INstruments-based methodological quality and measurement property assessment

A COnsensus-based Standards for the selection of health Measurement INstruments (COSMIN)-based appraisal was conducted for each included study and reported measurement property. Methodological quality was rated using the applicable COSMIN Risk of Bias standards as very good, adequate, doubtful, or inadequate, and the overall rating for each study-property combination was determined using the worst-score count principle.^19^ Results for each measurement property were then judged against COSMIN criteria for good measurement properties and classified as Sufficient, Insufficient, or Indeterminate; a dash (-) was used when a property was not reported or was not applicable.^20^ In the summary table, cell text indicates the result judgment, whereas cell fill indicates methodological quality. Because this review included performance-based, observational, self-report, and environmental instruments, COSMIN principles were applied to the relevant properties reported for each instrument, and ratings were assigned only to properties directly evaluated in the original studies.^19^^,^^20^

## Results

### Study selection

**Fig 1:**
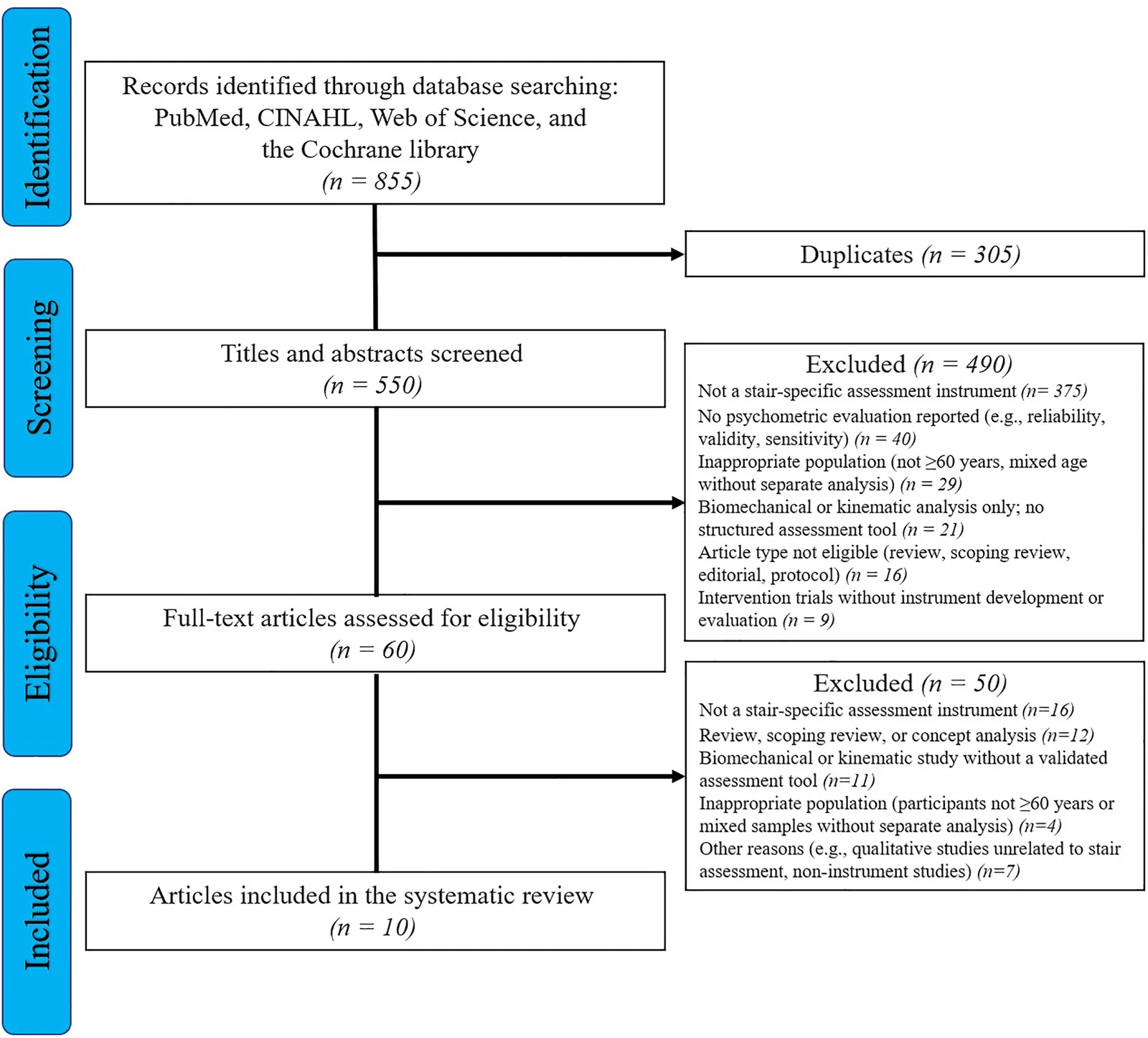
PRISMA 2020 flow diagram of study selection.

After deduplication, 855 records were screened by title and abstract, 60 full-text articles were assessed for eligibility, and 10 studies were included in the review (fig 1).

All findings summarized below are presented in supplemental table S1, which provides the full characteristics of the included studies, stair-related instruments, and reported measurement properties and key results for each instrument. To improve readability in the main text, the detailed evidence table was moved to the supplemental material.

### Study characteristics

The 10 included studies reported data from 1778 participants, with sample sizes ranging from 32 to 513 participants. Mean participant ages ranged from 61.4 to 82.7 years, and reported ages ranged from 65 to 97 years. Studies were conducted across community-dwelling, clinical, outpatient rehabilitation, and research-based settings. Study designs included cross-sectional reliability and validity studies, validation studies, and prospective longitudinal cohort studies examining prediction, responsiveness, and thresholds for change. The included populations comprised older adults with unilateral total knee arthroplasty, symptomatic knee osteoarthritis, chronic stroke, and risk for mobility decline, as well as community-dwelling samples without disability or dementia. Sex distribution was inconsistently reported across studies.

### Stair-related instruments identified

Across the included studies, the stair-related instruments included performance tests, 1 clinician-rated observational tool, and self-report measures of stair self-efficacy. Most instruments were performance-based stair or step tests, including the 11-step Stair Ascend/Descend Test total time (STTotal-11), Stair Up Test (STUp-11), 15-second Step Up and Down Test (StUD), Five-Step Test (FST), 4-Step Stair Climb Power Test (4SCPT), 3-step Stair Negotiation Time test, and the 6-step timed Stair Climb Test. The review also identified STEPS as a clinician-rated observational assessment and 2 self-report measures: the translated version of the Stair Self-Efficacy Questionnaire and the Stair Self-Efficacy scale.

### Measurement properties

Across instruments, the most frequently evaluated measurement properties were reliability and construct validity. In contrast, measurement error, responsiveness, predictive validity, internal consistency, and structural validity were examined less consistently. Evidence for criterion, predictive, and cross-cultural or structural validity was limited.

### Reliability

Reliability evidence was strongest for the performance-based and observational measures. The STEPS demonstrated strong reliability, with interrater reliability for the total score of an intraclass correlation coefficient (ICC)=0.91 (95% CI, 0.85-0.95), interrater reliability for ascent and descent subcomponents of ICC=0.83 and 0.91, respectively, and intrarater reliability ranging from ICC=0.91 to 0.93. The STTotal-11 and STUp-11 also showed strong interrater reliability (ICC=0.94 and 0.93, respectively). For the StUD, test-retest reliability was high, and the FST and 4SCPT also demonstrated sufficient reliability in their respective populations. By contrast, reliability evidence for the self-report stair self-efficacy measures was less consistent, with internal consistency and test-retest reliability generally judged as indeterminate in the COSMIN summary. For internal consistency, these indeterminate judgments should be interpreted in relation to the absence or insufficiency of evidence of structural validity, because COSMIN recommends interpreting internal consistency only after the scale’s dimensionality has been adequately evaluated.

### Validity

Construct validity was the most examined aspect of validity. The STEPS showed moderate-to-strong associations with established measures of mobility and lower-extremity performance, including the Timed Up and Go and 5-times sit-to-stand, and demonstrated known-groups validity by differentiating participants with and without recent falls. Convergent validity was also supported for the STTotal-11, STUp-11, StUD, FST, 4SCPT, and 6-step timed Stair Climb Test, primarily through associations with physical performance, mobility, strength, or functional status measures. In contrast, evidence for the validity of the self-report stair self-efficacy instruments was more limited, with construct validity generally remaining indeterminate. Structural validity was reported only for the translated version of the Stair Self-Efficacy Questionnaire and was judged indeterminate.

### Predictive validity and responsiveness

Evidence for predictive validity was limited and should be distinguished from criterion validity. The strongest support came from the 3-step stair-negotiation time test, in which slower stair-negotiation time predicted subsequent functional decline in community-dwelling older adults. In the COSMIN summary, predictive validity for this instrument was judged Sufficient. Responsiveness, defined as the ability of an instrument to detect meaningful change in stair performance over time, such as improvement or decline after an intervention or during follow-up, was supported for the StUD and for the 3-step stair-negotiation time test in the longitudinal responsiveness analysis. In contrast, the responsiveness of the 6-step timed Stair Climb Test was judged Insufficient because only 2 of 5 predefined hypotheses were met, indicating preliminary but inconsistent evidence for responsiveness in adults with hip or knee osteoarthritis.

### Measurement error, interpretability, and thresholds for change

Measurement error was reported for several performance-based instruments, including the STTotal-11, STUp-11, StUD, FST, 4SCPT, 3-step stair-negotiation time test, and the 6-step timed Stair Climb Test. However, in the COSMIN-based summary, measurement error was generally judged as Indeterminate, largely because indices such as the standard error of measurement, the minimum detectable change, or related statistics were reported without a clear criterion for adequacy. For the 3-step stair-negotiation time test, clinically meaningful change thresholds were derived using distribution- and anchor-based methods, supporting its interpretability for longitudinal change.

### COSMIN-based methodological quality and measurement property assessment

Table 1 summarizes the COSMIN-based methodological quality ratings and measurement property judgments for the included stair-related instruments. Overall, the psychometric evidence was heterogeneous, and no instrument demonstrated complete evaluation across all measurement properties.

**Table 1:** Methodological quality and measurement property assessment

Measurement Tool	Population	Reliability			Validity					
		Internal Consistency	Reliability	Measurement Error	Content Validity	Structural Validity	Construct Validity	Criterion Validity	Predictive Validity	Responsiveness
STTotal-11 (Almeida et al, 2010)^21^	Unilateral TKA, 2-6 months post-op		Sufficient^++^	Indeterminate^++^			Sufficient^++^			
STUp-11 (Almeida et al, 2010)^21^	Unilateral TKA, 2-6 months post-op		Sufficient^++^	Indeterminate^++^			Sufficient^++^			
StUD test (Almeida et al, 2021)^22^	Symptomatic KOA		Sufficient^++^	Indeterminate^++^			Sufficient^++^			Sufficient^+^
SSE questionnaire, German (Altmeier et al, 2018)^23^	Community-dwelling older adults	Indeterminate^++^	Indeterminate^+^			Indeterminate^++^	Indeterminate^++^			
Stair Self-Efficacy scale (Hamel & Cavanagh, 2004)^24^	Community-dwelling adults aged 75+	Indeterminate^++^					Indeterminate^++^			
STEPS (Kegelmeyer et al, 2024)^15^	Community-dwelling older adults aged 65+		Sufficient^+++^	Indeterminate^+^	Indeterminate^+^		Sufficient^++^			
Five-Step Test (Ng et al, 2018)^25^	People with chronic stroke aged >55 years		Sufficient^+++^	Indeterminate^++^			Sufficient^++^			
4SCPT (Ni et al, 2017)^26^	Community-dwelling older adults at risk for mobility decline		Sufficient^++^	Indeterminate^++^			Sufficient^++^	Indeterminate^+^		
3-step stair negotiation time test (Oh-Park et al, 2011)^13^	Community-dwelling nondisabled older adults aged 70+		Indeterminate^+^		Indeterminate^+^		Indeterminate^++^		Sufficient^+++^	
3-step stair negotiation time test (Oh-Park et al, 2012)^27^	Community-dwelling older adults, 1-year follow-up			Indeterminate^++^						Sufficient^++^
6-step timed Stair Climb Test (Sharma et al, 2022)^28^	Adults with hip or knee osteoarthritis		Sufficient^++^	Indeterminate^++^			Sufficient^++^			Insufficient^++^

Legend: Risk of Bias (ROB), +++ = Very good ROB, ++ = Adequate ROB, + = Doubtdul ROB, - = Inadequate ROB.

Reliability was the most consistently supported property. Sufficient reliability was identified for the STTotal-11, STUp-11, StUD, STEPS, FST, 4SCPT, and 6-step timed Stair Climb Test. Methodological quality for reliability was most often adequate, and reached very good for selected instruments in table 1.

Construct validity was also supported for several performance-based and observational tools, including the STTotal-11, STUp-11, StUD, STEPS, FST, 4SCPT, and the 6-step timed Stair Climb Test. In contrast, the self-report stair self-efficacy measures showed more limited psychometric support, with internal consistency, structural validity, and construct validity largely rated as indeterminate.

Measurement error was most often judged Indeterminate across instruments. Responsiveness was judged Sufficient for the StUD and the 3-step stair-negotiation time test assessed longitudinally, but Insufficient for the 6-step timed Stair Climb Test because only 2 of 5 predefined responsiveness hypotheses were met. Predictive validity was supported only for the 3-step stair-negotiation time test. Overall, the updated findings indicate that the strongest psychometric support is currently available for selected performance-based stair tests and the STEPS observational assessment, whereas evidence remains incomplete for most other instruments, particularly self-report measures.

## Discussion

### Interpretation in the context of existing literature

Findings from this review highlight the distinction between stair-specific assessments and general fall-risk tools used in rehabilitation practice. Although common balance and mobility measures have established psychometric value, they primarily reflect level-ground performance and may not capture the biomechanical, sensory, and attentional demands of stair negotiation. The instruments identified in this review therefore address stair-specific constructs, including ascent and descent time, step capacity, movement quality, lower-limb power, and stair-related confidence.^10^^,^^11^^,^^29^

The predominance of performance-based stair assessments in this review also aligns with clinical demand for brief, low-cost, and feasible tools. Timed stair tests have been used across clinical and research settings because stairs are readily available and performance can be quantified with minimal equipment, although protocol variability remains a challenge.^30^^,^^31^ In the present review, psychometric evidence was strongest for reliability and construct validity. Sufficient reliability was identified for STTotal-11, STUp-11, StUD, STEPS, FST, 4SCPT, and the 6-step timed Stair Climb Test, whereas sufficient construct validity was identified for STTotal-11, STUp-11, StUD, STEPS, FST, 4SCPT, and the 6-step timed Stair Climb Test. These findings suggest that timed and observational stair-based measures can provide clinically useful information about stair-specific functional performance, particularly when the purpose is to characterize mobility demands not fully reflected by level-ground tests.^15^^,^^21^^,^^22^^,^^25^^,^^26^^,^^28^

Despite these favorable findings, evidence for predictive validity, responsiveness, and measurement error remained limited across most instruments. In the COSMIN-based summary, responsiveness was supported for the StUD and the 3-step stair-negotiation time test, whereas the 6-step timed Stair Climb Test showed preliminary but inconsistent evidence of responsiveness in adults with hip or knee osteoarthritis. Predictive validity was supported only for the 3-step stair-negotiation time test. Measurement error was frequently judged indeterminate because studies often reported standard error of measurement, minimal detectable change, smallest detectable change, or related indices without a clear threshold for judging adequacy. These gaps are clinically important because decisions about monitoring change, estimating prognosis, or supporting longitudinal decision-making require more than cross-sectional reliability and construct validity alone.^13^^,^^27^^,^^32^

The review also suggests that self-report stair self-efficacy measures may provide information not captured by observed stair performance alone. Stair self-efficacy reflects perceived capability and confidence during stair negotiation, which may influence activity avoidance, cautious behavior, and real-world participation among older adults.^23^^,^^24^^,^^33^ However, compared with performance-based and observational tools, psychometric support for the self-report measures identified in this review was less complete. Internal consistency, structural validity, and construct validity were generally indeterminate, indicating that these tools may be conceptually relevant but currently less well established for clinical decision-making. Specifically, the indeterminate internal consistency ratings should not be interpreted as evidence of poor reliability. Rather, they reflect insufficient evidence regarding structural validity, which limits interpretation of whether the items form a sufficiently unidimensional scale.

### Clinical and research implications

From a clinical perspective, these findings support the use of stair-specific instruments as complementary components of a multifactorial fall-risk evaluation rather than as stand-alone tools. This interpretation is consistent with guidance recommending that fall risk be assessed through a broader evaluation of interacting factors rather than inferred from any single mobility measure.^5^^,^^34^^,^^35^ Brief performance-based stair tests are likely the most defensible options for routine use because they are feasible to administer and currently have the strongest evidence for reliability and construct validity. Among these, the 3-step stair-negotiation time test may be especially useful when prognosis or repeated monitoring is needed, as longitudinal evidence supports its predictive validity and the use of clinically interpretable change thresholds in older adults.^13^^,^^30^^,^^31^^,^^26^^,^^27^ Clinician-rated observational tools such as STEPS may also be useful for capturing movement quality, safety behaviors, and compensatory strategies during stair use. The sufficient content validity rating for STEPS supports the relevance of its stair-related content, but current evidence remains incomplete across other measurement properties needed for prognosis or longitudinal monitoring.

However, the current evidence base does not support relying on most stair-related instruments alone for prognosis or for monitoring change over time. No single instrument demonstrated a complete psychometric evaluation across all relevant measurement properties, and several tools had evidence largely limited to reliability and construct validity. Accordingly, stair-specific measures are best interpreted alongside broader assessment of gait, balance, strength, cognition, and confidence, consistent with prior work showing that no single functional mobility tool can predict falls with high certainty across settings.^4^^,^^5^^,^^9^^,^^36^

Self-report stair self-efficacy measures may still be useful as adjuncts in cases where observed stair performance does not fully explain the difficulty or perceived risk experienced by the individual. These measures offer a low-burden way to assess confidence and perceived safety during stair negotiation, and may help clinicians identify behavioral avoidance or fear-related limitation that would not be evident from timed performance alone.^33^ However, based on the current review, these instruments should be interpreted with caution until stronger evidence is available regarding their structural validity, reliability, and construct validity in older adult populations.

At the research and reporting levels, substantial heterogeneity in stair-test protocols, including the number of steps, stair geometry, pace instructions, handrail rules, and outcome definitions, likely contributed to between-study variability and limited direct comparability across instruments. This concern is consistent with the broader stair-negotiation literature, which shows that changes in architecture, visual demands, and handrail use can meaningfully alter task demands and movement strategies.^1^^,^^10^^,^^29^ This heterogeneity supports the use of narrative synthesis in the present review and underscores the need for more standardized stair-task protocols and reporting practices, consistent with PRISMA 2020 and PRISMA-COSMIN reporting guidance.^37^^,^^38^ Future studies should more consistently evaluate measurement error, responsiveness, predictive validity, and content validity, and should report thresholds that support interpretation of change over time.

### Study limitations

Several limitations should also be considered when interpreting the findings of this review. First, the evidence base was limited to 10 included studies, with substantial heterogeneity across study designs, participant characteristics, clinical contexts, instruments, outcome measures, and stair-assessment protocols. Differences in the number of steps, stair geometry, pace instructions, handrail conditions, and reported measurement properties reduced comparability across studies; therefore, meta-analysis was not possible, and findings were synthesized narratively. Second, the identified instruments reflected distinct assessment domains, including performance-based stair tasks, observational rating of stair performance, and self-reported stair self-efficacy, and thus should not be interpreted as equivalent indicators of a single underlying construct. Third, the psychometric evaluation was incomplete across instruments. Reliability and construct validity were the most frequently examined properties, whereas measurement error was often indeterminate, and responsiveness, predictive validity, internal consistency, and structural validity were reported less consistently. No instrument had evidence spanning all major measurement properties. Fourth, the search strategy focused on general stair-related terminology and did not include specific architectural subtypes, such as spiral staircases, which may have limited the retrieval of studies focused on specialized stair designs. Scopus was also not included among the databases searched, which may have limited the retrieval of relevant studies indexed outside PubMed, CINAHL, Web of Science, and the Cochrane Library. Fifth, although study selection was conducted independently by 2 reviewers, the possibility of selection bias cannot be fully excluded. Finally, external validity may be limited because several studies were based on condition-specific populations rather than more broadly representative samples of older adults.

## Conclusion

Stair-related assessment instruments for older adults show promising but incomplete psychometric support. Current evidence most strongly supports selected performance-based stair tests and the STEPS observational assessment for use as complementary components of multifactorial fall-risk evaluation. However, no instrument has been comprehensively evaluated across all major measurement properties, and evidence remains limited for responsiveness, predictive validity, measurement error, internal consistency, and structural validity. Future research should use standardized stair protocols, include more diverse older adult populations, and evaluate clinically interpretable thresholds to guide instrument selection and monitoring over time.

## Disclosures

None.

## Appendix Supplementary materials

Image, application 1

Image, application 2

## Footnotes

Supplementary material associated with this article can be found, in the online version, at doi:10.1016/j.arrct.2026.100668.
