# Supplementary material for: A Systematic Review of Stair Fall Risk Assessment Instruments in Older Adults
Source: Arch Rehabil Res Clin Transl. 2026 Jun 28;8(3):100668. doi: 10.1016/j.arrct.2026.100668 (PMC13591183; doi:10.1016/j.arrct.2026.100668)
Supplement: Supplementary file 1 [file mmc1.docx]

**Appendix 1 Full Electronic Search Strategies for PubMed, CINAHL, Web of Science, and Cochrane Library**

The electronic search strategies were developed to identify studies evaluating stair-related fall-risk assessment instruments in older adults. Searches were conducted in PubMed, CINAHL, Web of Science, and Cochrane Library from database inception through October 2025. Search terms combined controlled vocabulary, when available, and free-text keywords related to stairs, stair negotiation, falls, fall risk, assessment instruments, and older adults. Search syntax was adapted for each database according to its indexing system and search interface. Reference lists of included studies and relevant review articles were also screened manually to identify additional eligible studies.

**PubMed (searched October, 2025)**

(“stairs”[MeSH Terms] OR stair*[Title/Abstract] OR “stair negotiation”[Title/Abstract] OR “stair climbing”[Title/Abstract] OR step*[Title/Abstract])

AND

(“accidental falls”[MeSH Terms] OR fall*[Title/Abstract] OR “fall risk”[Title/Abstract])

AND

(“risk assessment”[MeSH Terms] OR assess*[Title/Abstract] OR instrument*[Title/Abstract] OR test*[Title/Abstract] OR measure*[Title/Abstract] OR screening[Title/Abstract])

AND

(“aged”[MeSH Terms] OR older adult*[Title/Abstract] OR elderly[Title/Abstract] OR older people[Title/Abstract])

**CINAHL via EBSCOhost (searched October, 2025)**

(stair OR stairs OR staircase OR stairway OR “stair negotiation”)

AND fall*

AND (risk OR prediction OR screen* OR assess*)

AND (tool OR instrument OR scale OR test OR measure OR assessment)

AND (“older adult*” OR elderly OR aged)

**Web of Science Core Collection (searched October, 2025)**

TS=(stair* OR “stair negotiation” OR “stair climbing”)

AND

(fall* OR “fall risk”)

AND

(“risk assessment” OR screening OR assess* OR predict*)

AND

(instrument* OR tool* OR scale* OR test*)

AND

(“older adult*” OR elderly OR aged)

**Cochrane Library (searched October, 2025)**

(stair OR stairs OR staircase OR stairway OR “stair negotiation”):ti,ab,kw

AND fall*:ti,ab,kw

AND (risk OR prediction OR screen* OR assess*):ti,ab,kw

AND (tool OR instrument OR scale OR test OR measure OR assessment):ti,ab,kw

AND (“older adult*” OR elderly OR aged):ti,ab,kw
